# Comparison of Intravenous Ondansetron and Dexamethasone for Preventing Propofol-Induced Pain During Laparoscopic Cholecystectomy: A Double-Blind, Randomized Study

**DOI:** 10.7759/cureus.67382

**Published:** 2024-08-21

**Authors:** Aravind Aditya, Khushboo Pandey, Manisha Bhagat, Wasimul Hoda, Priyanka Oraon, Ladhu Lakra

**Affiliations:** 1 Anesthesiology, Rajendra Institute of Medical Sciences, Ranchi, IND; 2 Anesthesiology, All India Institute of Medical Sciences, Rajkot, Rajkot, IND

**Keywords:** laparoscopic cholecystectomy, pretreatment, ondansetron, dexamethasone, propofol induced pain

## Abstract

Background

Propofol is the most common induction agent used in current anesthesia practice. Patients receiving propofol injections commonly experience varying degrees of pain, creating an unpleasant anesthesia experience.

Methods

Seventy-two patients, aged between 18 and 70, scheduled for elective laparoscopic cholecystectomy under general anesthesia were randomized into two groups. Group D received 8 mg of dexamethasone, and Group O received 8 mg of ondansetron intravenously before induction. After five seconds, mid-arm venous occlusion was applied for one minute using a tourniquet. Propofol (0.5 mg/kg) was administered intravenously over five seconds, and patients rated the injection pain over the next 15 seconds. The primary outcome was pain intensity using the Verbal Rating Scale during propofol injection. Secondary outcomes included intraoperative hemodynamic changes and postoperative nausea and vomiting (PONV). Normally distributed variables were compared using the Student's t-test, non-normally distributed variables using the Mann-Whitney U-test, and qualitative data using the chi-square or Fisher’s exact test. Statistical significance for the study was set at p < 0.05.

Results

In Group D, 30 out of 36 patients (83.3%) experienced no pain, while four patients (11.1%) reported mild pain, two patients (5.6%) reported moderate pain, and no patients (0.0%) reported severe pain. In contrast, in Group O, only 15 out of 36 patients (41.6%) experienced no pain, with 12 patients (33.3%) experiencing mild pain, seven patients (19.4%) experiencing moderate pain, and two patients (5.6%) experiencing severe pain. Overall, six out of 36 patients in Group D (16.7%) experienced some level of pain, compared to 21 out of 36 patients in Group O (58.3%), a statistically significant difference (p < 0.05). Regarding postoperative nausea, 16 out of 36 patients in Group Dexamethasone (44.44%) experienced nausea, whereas 23 out of 36 patients in Group Ondansetron (63.88%) reported this symptom, with the difference being statistically significant (p = 0.0372). Additionally, postoperative vomiting occurred in nine out of 36 patients in Group Dexamethasone (25%), compared to 18 out of 36 patients in Group Ondansetron (50%), with this difference also reaching statistical significance (p= 0.026).

Conclusions

Intravenous dexamethasone before propofol administration reduces injection pain and PONV in laparoscopic cholecystectomy more effectively as compared to ondansetron.

## Introduction

Propofol is commonly used as an intravenous induction agent for general anesthesia. It is preferred for daycare surgeries and total intravenous anesthesia due to its rapid onset and recovery. However, pain at the injection site remains a common side effect of propofol, which not only impacts patient satisfaction but can also trigger significant cardiovascular responses, including myocardial ischemia in high-risk patients. These responses are driven by the acute pain from the injection, potentially causing severe hemodynamic changes that may precipitate ischemic events in vulnerable individuals [1]. The high incidence of propofol injection pain (POPI) reported in the literature ranges from 28% to 90% in adults and 28-85% in children, highlighting the need for effective pain mitigation strategies [2,3]. Various methods, including pharmacological interventions with intravenous lidocaine, fentanyl, alfentanil, meperidine, metoclopramide, and non-pharmacological measures like cooling the extremity or altering drug formulations, have been mentioned with varying mixed results [4,5].

Dexamethasone, recognized for its anti-inflammatory properties and ability to reduce postoperative pain and nausea, offers potential relief for POPI [6]. Beyond its anti-inflammatory effects, dexamethasone stabilizes cell membranes and inhibits inflammatory mediators, reducing peripheral nociception. It also suppresses prostaglandins and cytokines, thereby lowering postoperative pain sensitivity. Whereas ondansetron, a 5-HT3 receptor antagonist with sodium channel blocking capabilities, may offer superior local anesthetic effects compared to lidocaine, further supporting its use in reducing POPI [7,8]. In this study, dexamethasone and ondansetron were chosen for their dual benefits in managing both POPI and postoperative nausea and vomiting (PONV). It was hypothesized that both dexamethasone and ondansetron would not only reduce nausea and vomiting but also alleviate POPI, thereby improving patient experience, facilitating faster recovery, and reducing postoperative costs. However, to our knowledge, no prior studies have compared the effectiveness of dexamethasone and ondansetron in managing POPI and preventing PONV in patients undergoing laparoscopic cholecystectomy.

Hence, this study aims to evaluate the efficacy of dexamethasone and ondansetron in mitigating pain associated with propofol injections and preventing PONV in patients undergoing laparoscopic cholecystectomies. The primary outcome was a reduction in pain intensity using the Verbal Rating Scale (VRS) after propofol injection. Secondary outcomes included intraoperative hemodynamic changes and PONV in the postoperative period.

## Materials and methods

This double-blinded, randomized clinical trial study was prospectively registered with the Clinical Trials Registry - India. Ethical clearance was obtained from the Institutional Ethics Committee (approval number 184/IEC dated April 1, 2021). The data for this study was collected in a tertiary-level hospital setting in Jharkhand state. The recruitment and follow-up periods spanned 1.5 years from the time of approval by the research and ethics committee. The study adhered to the principles outlined in the Declaration of Helsinki (2013), ensuring compliance with ethical standards for medical research involving human subjects.

After written informed consent, 72 patients scheduled for laparoscopic cholecystectomy aged between 18 and 70 years of either gender, belonging to the American Society of Anesthesiologists (ASA) physical status (PS) 1 and 2, were recruited. Patients not willing to give consent, known hypersensitivity to the study drug, history of motion sickness, history of PONV, past history of chemotherapy or radiotherapy, laparoscopic surgeries that were converted to open surgeries, and if drugs other than ondansetron or dexamethasone were used for alleviation of pain on propofol injection were excluded from the study.

Patients were kept at nil per oral for six hours for solids and two hours for clear liquids preoperatively. In the operating room, standard monitors were attached, and a 20G intravenous cannula was placed without using any local anesthetic on the dorsum of the hand. Subsequently, a Ringer’s lactate infusion was initiated. Baseline hemodynamic parameters, including heart rate (HR), mean blood pressure, and oxygen saturation, were recorded at this initial time point as preinjection vitals.

The enrolled patients were assigned into two groups based on the computer-generated random number table. Allocation was concealed using opaque sealed envelopes, which were opened at the time of drug preparation by a senior anesthesiologist not involved in the study. A total volume of 4 ml of a clear solution of the pretreatment drug was prepared and administered by the same anesthesiologist to ensure blinding. The patient and the investigator were blinded by group allocation.

Group D patients received 8 mg of dexamethasone (2 ml) with 2 ml of 0.9% normal saline (total 4 ml), and Group O patients received 8 mg (4 ml) of Ondansetron. After five seconds, mid-arm venous occlusion was done for one minute using a tourniquet. Subsequently, 1% propofol (Neorof (R) 1%, Neon Laboratories Ltd., Mumbai, India) was drawn immediately before use in a 10 ml syringe, and 0.5 mg/kg of it was given over five seconds. During the next 15 seconds, the patients were asked to rate the pain after a propofol injection. The intensity of pain they experienced was then graded using a VRS, where 0 stood for no pain, 1 for mild pain, 2 for moderate pain, and 3 for severe pain [9]. Detailed information regarding the VRS score, which is based on the degree of pain and patient response, is provided in Appendix A. The remainder of the calculated propofol dose was given, and induction of anesthesia was continued.

General anesthesia was continued with rocuronium (0.8 mg/kg), followed by intubation, and maintained on air, oxygen, and isoflurane. Post-injection, pre-intubation, and post-intubation hemodynamic parameters such as HR, mean arterial pressure (MAP), and oxygen saturation were noted. Injections of neostigmine and glycopyrrolate were used to reverse anesthesia at the conclusion of surgery. The patients were then followed up on PONV for a period of 24 hours postoperatively. During the intraoperative period, all patients received 1 g of intravenous paracetamol just after induction. Postoperatively, in accordance with institutional protocol, 1 g of intravenous paracetamol every eight hours and 75 mg of diclofenac in 100 ml of normal saline every 12 hours were administered slowly for effective pain management.

The incidence and severity of PONV were evaluated using a standardized scoring system that categorizes PONV into four levels: 0 (no nausea or vomiting), 1 (nausea only), 2 (vomiting once), and 3 (vomiting more than once). All patients in the study were considered high risk for PONV, ensuring a consistent and rigorous approach to assessment and management. For severe PONV, the first-line rescue medication was 10 mg of intravenous metoclopramide, administered as a stat dose and repeated as needed.

The primary objective was a comparison of intravenous ondansetron and dexamethasone as pretreatment drugs to prevent POPI in patients undergoing laparoscopic cholecystectomy. The secondary objective was to observe hemodynamic changes and the incidence of PONV after laparoscopic cholecystectomy.

Drawing from previous studies, it was estimated that the incidence of POPI is 56%, with a 24% reduction considered significant [10]. To achieve a power of 80% and maintain an alpha value of 0.05, our calculations indicated that a minimum of 33 participants per group was required. To accommodate potential dropouts, the sample size was adjusted to 36 participants per group.

The data were coded and entered into Microsoft Excel for the Microsoft 365 version (Microsoft Corporation, Redmond, WA, USA) for initial recording. Subsequent data analysis was conducted using IBM SPSS Statistics for Windows, Version 23.0 (Released 2015; IBM Corp., Armonk, NY, USA). The normality of the data distribution was evaluated using the Shapiro-Wilk test. Continuous variables, including age and weight, were analyzed using either the Student's t-test or the Mann-Whitney test, depending on the data distribution. Categorical variables were expressed as frequencies and percentages and were compared using the chi-square test or Fisher’s exact test, as appropriate. Statistical significance was determined for p-values less than 0.05.

## Results

**Figure 1:**
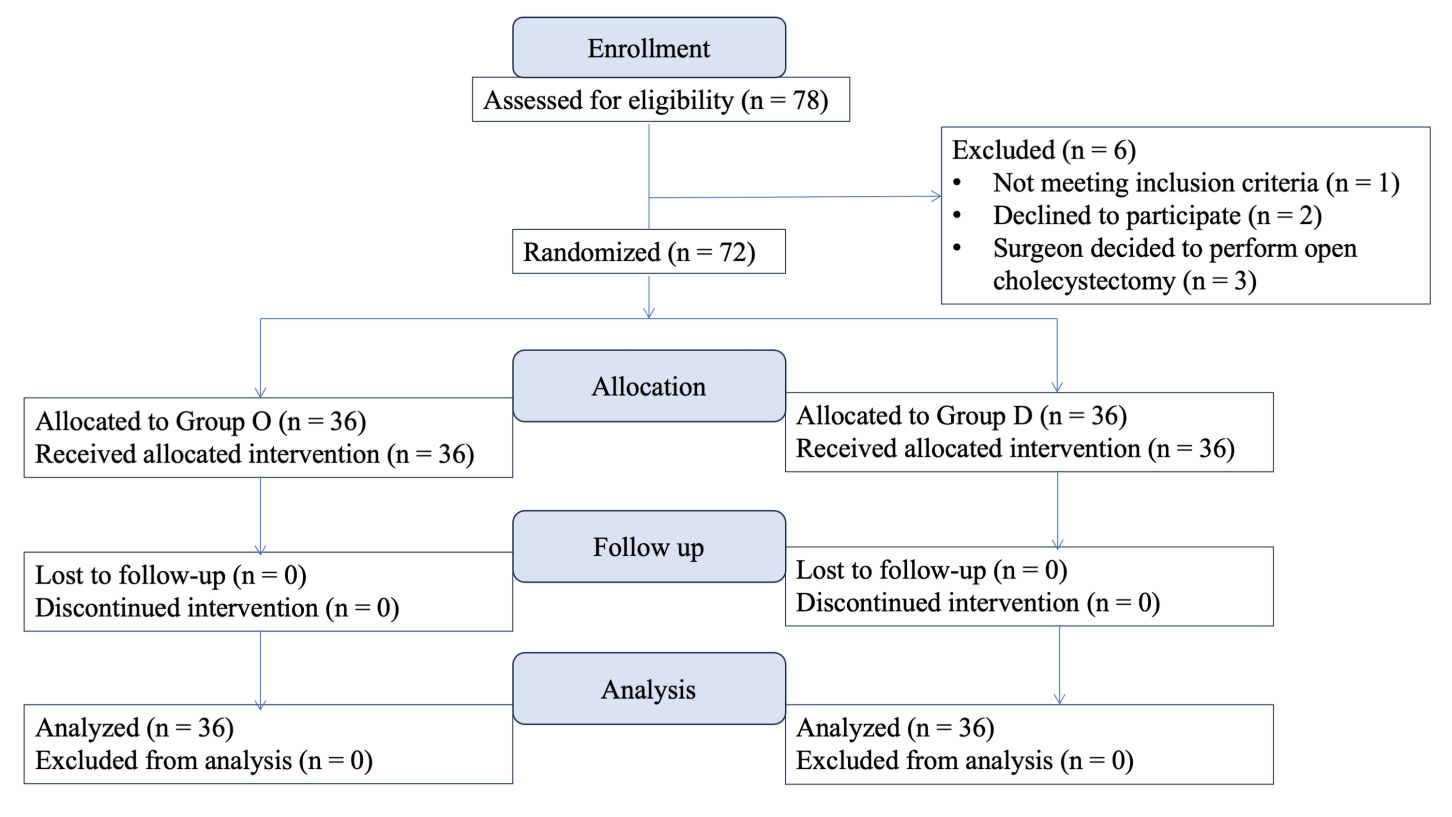
CONSORT diagram showing the flow of patients through various stages of the trial Group D: dexamethasone; Group O: ondansetron CONSORT: Consolidated Standards of Reporting Trial

The Consolidated Standards of Reporting Trials (CONSORT) flow diagram in Figure 1 depicts the progression of patients through the various stages of the randomized trial. Initially, 78 patients were assessed for eligibility, with six patients excluded from the study due to various reasons: one patient did not meet the inclusion criteria, two patients declined to participate, and three patients underwent conversion to open cholecystectomy, making them ineligible for the study protocol.

After these exclusions, 72 patients were successfully randomized, with 36 allocated to Group O (ondansetron) and 36 to Group D (dexamethasone). All patients in both groups received the allocated interventions as planned. During the follow-up phase, there were no instances of loss to follow-up or discontinuation of the intervention in either group, resulting in a complete dataset for final analysis.

The analysis included all 72 patients who completed the study, with 36 in Group O and 36 in Group D. No patients were excluded from the analysis, ensuring that the results accurately reflect the outcomes of all participants who were randomized and received the intervention.

The demographic characteristics of patients were comparable in both groups, as summarized in Table 1. The mean age was 38.9 ± 13.3 years in Group D and 39.2 ± 11.8 years in Group O. The sex distribution was 10 males/26 females in Group D and 6 males/36 females in Group O. Both groups had similar mean body weights, 57.8 ± 12.4 kg in Group D and 57.5 ± 10.8 kg in Group O. The ASA physical status distribution was also similar between the groups.

**Table 1:** Demographic data Values are expressed as mean ± SD. ASA PS: American Society of Anesthesiologists physical status

Patient characteristics	Group D (n = 36)	Group O (n = 36)	p-value
Age (years)	38.9 ± 13.3 years	39.2 ± 11.8 years	0.91
Sex (male/female)	10/26	6/36	0.26
Body weight (kg)	57.8 ± 12.4	57.5 ± 10.8	0.92
ASA PS 1/2	22/14	27/9	0.21

Table 2 presents the incidence of pain during propofol injection in Group D (n = 36) and Group O (n = 36), measured using the Visual Rating Scale (VRS). In Group D, 30 out of 36 patients (83.3%) reported no pain during the injection, while in Group O, only 15 out of 36 patients (41.6%) reported no pain. This difference was statistically significant (p < 0.05).

**Table 2:** POPI measured as VRS Values are expressed as numbers (percentages). * significant p-value POPI: propofol-induced pain; VRS: Verbal Rating Scale

VRS	Group D (n = 36)	Group O (n = 36)	p-value
0	30 (83.3)	15 (41.6)	0.00065*
1	4 (11.1)	12 (33.3)	0.0472*
2	2 (5.6)	7 (19.4)	0.154
3	0 (0.0)	2 (5.6)	0.473
Total patients experienced pain	6 (16.7)	21 (58.3)	0.00065*

Regarding mild pain (VRS score of 1), four out of 36 patients (11.1%) in Group D experienced mild pain, compared to 12 out of 36 patients (33.3%) in Group O, with a statistically significant difference (p < 0.05). For moderate pain (VRS score of 2), two out of 36 patients (5.6%) in Group D reported moderate pain, whereas seven out of 36 patients (19.4%) in Group O reported moderate pain.

In terms of severe pain (VRS score of 3), no patients in Group D (0.0%) experienced severe pain, while two out of 36 patients (5.6%) in Group O experienced it.

Overall, six out of 36 patients in Group D (16.7%) experienced some level of pain during the propofol injection, compared to 21 out of 36 patients (58.3%) in Group O. This overall difference in pain incidence between the two groups was statistically significant (p < 0.05).

**Figure 2:**
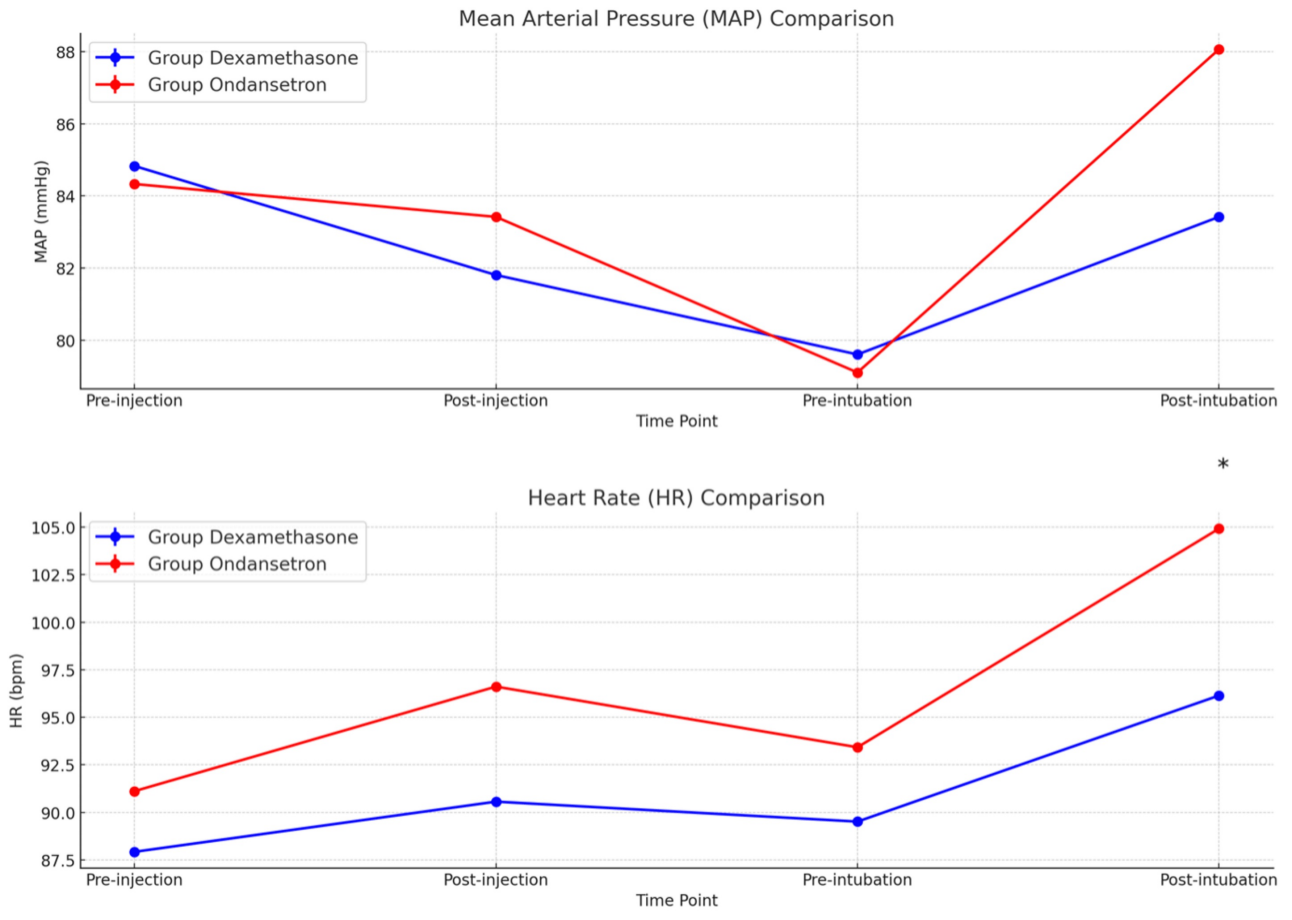
Line plot diagram showing a comparison of MAP and HR across time points between the dexamethasone and ondansetron groups Group Dexamethasone (blue line) and Group Ondansetron (red line) * Significant p-value (p = 0.0203) HR: heart rate; MAP: mean arterial pressure

Figure 2 provides a graphical line plot comparison of MAP and HR across various time points, highlighting the significant difference in post-intubation HR between the two groups. The hemodynamic parameters, including MAP and HR, were comparable between the two groups at all time points, except for post-intubation HR. As illustrated in Figure 2, the post-intubation HR was significantly lower in the dexamethasone group compared to the ondansetron group, with a p-value of 0.0203.

The incidence of PONV in two groups is shown in Table 3. The data indicate that the incidence of nausea was significantly lower in the Dexamethasone group, with 16 out of 36 patients (44.44%) experiencing nausea, compared to 23 out of 36 patients (63.88%) in the Ondansetron group. This difference was statistically significant, with a p-value of 0.0372.

**Table 3:** PONV PONV: postoperative nausea and vomiting

Parameter	Group Dexamethasone (n = 36)	Group Ondansetron (n = 36)	p-value
Nausea incidence (%)	16 (44.44%)	23 (63.88%)	0.0372
Vomiting incidence (%)	9 (25%)	18 (50%)	0.026

Similarly, the incidence of vomiting was also significantly lower in the Dexamethasone group, where nine out of 36 patients (25%) experienced vomiting, compared to 18 out of 36 patients (50%) in the Ondansetron group. This difference was statistically significant, with a p-value of 0.026.

No complications or adverse drug effects were observed throughout the study.

## Discussion

This was a prospective, randomized, double-blinded clinical trial study, carried out over a period of 18 months. The most common side effect of propofol is pain on injection, which is very distressing to patients and affects the overall quality of the anesthesia provided. The aim of this study was to compare the efficacy of intravenous dexamethasone and ondansetron as pretreatment drugs to prevent POPI in patients undergoing laparoscopic cholecystectomy, as well as prevent PONV up to 24 hours after the conclusion of surgery.

We incorporated dexamethasone in our study to alleviate POPI due to its biological rationale as well as its widespread therapeutic use, such as reduction of postoperative pain and alleviation of PONV [11]. Regarding the alleviation of POPI, the release of nitric oxide (NO) from the vessel wall during propofol injection is linked to the development of pain on injection, which is altered by corticosteroids such as dexamethasone [12,13].

Another proposed mechanism of POPI is described as immediate discomfort following propofol injection due to local irritation of the vessel wall, leading to activation of kallikrein and bradykinin, where ondansetron is postulated to effectively act due to its local anesthetic action [7]. Additionally, venous occlusion with tourniquet application following ondansetron administration has been found to enhance its local anesthetic action [14]. Therefore, we utilized ondansetron with venous occlusion using a tourniquet to alleviate POPI, in addition to its antiemetic action in laparoscopic surgeries, which had a higher anticipated risk of PONV.

This study showed that pretreatment with dexamethasone is more efficacious in reducing the incidence and intensity of POPI. The incidence of POPI in our study was 16.7% and 58.3% after pretreatment with dexamethasone and ondansetron, respectively.

The effect of intravenous dexamethasone on POPI was studied by Ahmad et al., who concluded that, although dexamethasone is frequently used as an antiemetic, it also provides prolonged postoperative analgesia and alleviates pain induced by propofol injection, primarily due to its attenuation of NO release [10]. These findings have been confirmed in the present study, highlighting the effectiveness of dexamethasone in reducing POPI and improving the overall quality of perioperative recovery.

Zahedi et al. conducted a study to evaluate the effectiveness of ondansetron vs. tramadol vs. saline as pretreatment drugs for the alleviation of POPI [15]. The effect of ondansetron on reducing POPI was superior as compared to the placebo. This is consistent with the results of our study, suggesting that pretreatment with ondansetron will be effective in reducing the incidence of pain on propofol injection, in addition to preventing PONV.

The mean verbal pain score was statistically reduced with dexamethasone as a pretreatment drug for propofol injection. Additionally, the verbal pain score was 0 for the majority of study subjects in the dexamethasone group (83.3%) as compared to the ondansetron group (41.6%). This could be justified by the significant attenuation of NO release with dexamethasone, which is a notable mechanism of POPI, as demonstrated by Adinehmehr et al., where the authors compared granisetron with dexamethasone for intravenous propofol pain [16]. These findings are also in correlation with a study by Sharma et al., where the effect of granisetron and dexamethasone was compared in patients undergoing laparoscopic gynecological surgeries for the prevention of PONV [17].

The MAPs in the dexamethasone group were comparable to those in the ondansetron group. The mean post-injection HRs were significantly lower in the dexamethasone group, and the intubation response to HR was also less pronounced in the dexamethasone group. This could be attributed to the anti-inflammatory effect of dexamethasone and the attenuation of the intubation response seen with corticosteroid administration. These findings are in line with a study by Sharma et al., where intraoperative HRs were recorded with dexamethasone vs. saline in patients undergoing spine surgeries under general anesthesia [18].

PONV can lead to patient dissatisfaction, prolong recovery, and increase perioperative care costs. Approximately one-third of surgical patients may be affected by PONV, with the incidence rising to as high as 70% among high-risk patients.

The prophylactic effect of dexamethasone on PONV has already been extensively demonstrated in a wide range of surgeries [19]. The utility of 5-HT3 receptor antagonists to inhibit nausea and vomiting has also been well described, which may be in part due to their local anesthetic properties and activity in the gut and/or vomiting center [20]. This study demonstrated that 16 subjects developed nausea in the dexamethasone group as opposed to 23 subjects in the ondansetron group, with the difference being statistically significant (p = 0.03). Similarly, the difference in the incidence of postoperative vomiting was also statistically significant in our study, i.e., 25% in the dexamethasone group vs. 50% in the ondansetron group (p = 0.02). This could be attributed to the timing of administration of dexamethasone and ondansetron, which was at the time of induction in our study. Most of our surgeries lasted approximately two hours, which coincides with the peak antiemetic effect of dexamethasone. Additionally, it is a long-acting antiemetic with an appreciable effect for up to 24 hours. Ondansetron, however, is a shorter-acting antiemetic with a peak effect at 0.5 to two hours and a half-life of three to four hours. This could possibly explain the relatively high incidence of PONV in the ondansetron group. Our findings are consistent with those of Arslan et al., where prevention of PONV was examined with propofol and dexamethasone vs. metoclopramide in patients undergoing laparoscopic cholecystectomy, demonstrating a similar frequency of postoperative vomiting in the dexamethasone group [21].

Another study conducted by Wang et al., regarding the prevention of PONV, deciphered a significant reduction in the incidence of PONV in the dexamethasone group as opposed to the control, substantiating the notable role of dexamethasone in the prevention of PONV in line with the findings of our study [22].

Based on the results, we suggest that dexamethasone can be as feasibly utilized as ondansetron in routine practice for the prevention of POPI and PONV in laparoscopic surgeries, with the additional advantage of better analgesia and perioperative recovery.

This study has a few limitations. Firstly, it is a single-center study, which may limit the generalizability of the findings. Additionally, the results should be further evaluated by including subjects with higher ASA grades to ensure broader applicability. Moreover, the study protocol for assessing POPI involved the administration of sub-anesthetic doses of propofol, which could potentially affect patients’ ability to accurately assess and report pain scores.

## Conclusions

Intravenous dexamethasone and ondansetron are effective pretreatment options for reducing the incidence and severity of POPI and PONV in patients undergoing laparoscopic cholecystectomy. Dexamethasone, in particular, is beneficial as a pretreatment due to its ability to lower verbal pain scores during propofol injection, maintain stable intraoperative hemodynamics, significantly prevent PONV, and enhance the overall quality of perioperative recovery.

## Appendices

Appendix A

**Table 4:** VRS score used in the study VRS: Verbal Rating Scale

Pain score	Degree of pain	Response
0	None	Negative response to questioning
1	Mild	Pain reported in response to questioning only without any behavioral signs
2	Moderate	Pain reported in response to questioning and accompanied by a behavioral sign or pain reported spontaneously without questioning
3	Severe	Strong verbal response or response accompanied by facial grimacing, arm withdrawal, or tears
